# A dynamic duo: Copper metalloregulators and continuous-wave electron spin resonance spectroscopy

**DOI:** 10.1016/j.bpr.2026.100259

**Published:** 2026-03-19

**Authors:** Alysia Mandato, Sunil Saxena, Sharon Ruthstein

**Affiliations:** 1 Department of Chemistry, University of Pittsburgh, Pittsburgh, Pennsylvania; 2 Department of Chemistry and the Institute of Nanotechnology and Advanced Materials, Faculty of Exact Sciences, Bar Ilan University, Ramat-Gan, Israel

## Abstract

This review highlights the use of continuous-wave electron spin resonance (ESR) spectroscopy to investigate protein site-specific dynamics and metal coordination in bacterial copper metalloregulators. ESR provides direct insight into how metal binding influences conformational flexibility, revealing how similar proteins can employ distinct mechanisms of transcriptional regulation. Studies of the activator CueR and the repressor CsoR show that these systems use fundamentally different strategies to control gene expression. CueR functions as a stable dimer that activates transcription through metal-induced conformational changes rather than oligomeric transitions. In *E. coli*, Cu(I) binding rapidly increases dynamics in the DNA-binding domain, while in *Pseudomonas aeruginosa*, CueR exhibits a more gradual, DNA-dependent response that reflects adaptation to alternative copper resistance pathways. In contrast, CsoR exists in a dynamic equilibrium between dimeric and tetrameric states, with oligomerization playing a central role in its repressor mechanism. Small shifts in subunit exchange may fine-tune DNA binding and facilitate dissociation upon Cu(I) coordination. This review demonstrates how ESR spectroscopy can capture the subtle structural and dynamical differences that define metalloregulator function and highlight the diversity of bacterial strategies for maintaining metal homeostasis.

## Why it matters

This review places continuous-wave electron spin resonance (ESR) spectroscopy within the broader framework of biophysical approaches that link protein structure, dynamics, and function. ESR provides site-specific information on protein fluctuations and metal coordination, which can add insight to understanding how localized dynamical changes in different regions of a protein impact DNA interactions and metal sensing. Comparative analyses of the bacterial copper metalloregulators CueR and CsoR reveal how related systems can use distinct dynamical and oligomeric strategies to achieve regulatory specificity. These examples illustrate how biophysical tools can illuminate the energetic and structural landscapes that govern protein function, demonstrating that dynamic coupling and conformational heterogeneity are fundamental principles underlying transcriptional regulation across diverse systems.

## Introduction

Metals play crucial roles in biological systems, functioning as electron carriers, structural elements, and catalytic cofactors in a wide range of processes. Transition metals, including iron, manganese, cobalt, nickel, and copper, are particularly important because of their ability to switch between oxidation states, thereby mediating redox reactions in living systems (1). Copper ions, for example, are incorporated into metalloenzymes that support key biological functions including cytochrome oxidase and superoxide dismutase (2,3,4). Despite its importance, excess free copper ions can be harmful to cells.

In bacteria, Cu(I) ions may exist in cytoplasm due to the reducing environment provided by antioxidants, such as glutathione, ascorbate, and oxidoreductases. In particular, glutathione can directly reduce free Cu(II) and form a complex with Cu(I) to stabilize it. However, excess Cu(I) can deplete glutathione, leading to metal stress (5,6,7). Under aerobic conditions, Cu(I) can react with endogenous hydrogen peroxide to produce the hydroxyl and carbonate radicals via Fenton and Haber-Weiss-like reactions (8). These radicals have been implicated in widespread oxidative damage to cellular macromolecules (9), though the details of this damage remain debated (8,10,11,12).

Alternatively, there are other mechanisms of copper toxicity, particularly under anaerobic conditions. In the absence of reactive oxygen species (i.e., hydroxyl radicals), Cu(I) has been shown to inactivate iron-sulfur clusters in *E. coli* cells, leading to cell death (13). More generally, copper toxicity has also been linked to the inactivation of essential biochemical pathways like central carbon metabolism or nucleotide synthesis (14,15). Additionally, it has been suggested that Cu(I) triggers protein misfolding and aggregation under aerobic conditions (16), with a recent study confirming this effect under anaerobic conditions (17).

To maintain homeostasis in the presence of copper or other metal ions, bacterial cells have proteins that regulate the concentration of metal ions. Metalloregulatory proteins function by controlling the transcription of genes that mediate excess metal ion concentrations (1,18). These metal ion transcription factors have high-affinity coordination sites, often with the sensitivity high enough to detect one ion per cell. When the protein coordinates the metal ion, it triggers transcription to express proteins that can minimize the metal ion toxicity, such as transporters or oxidases.

**Figure 1:**
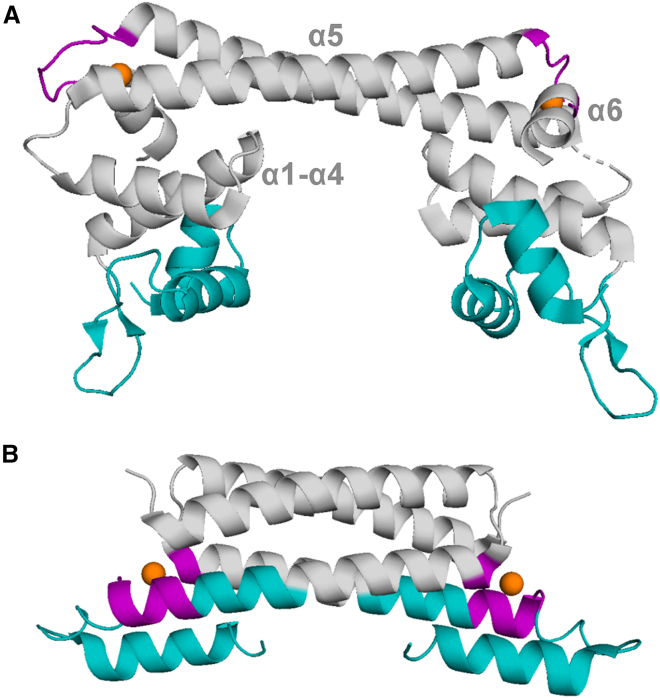
Illustrations of homodimeric structures of metalloregulator proteins. (A) *E. coli* CueR (PDB: 1Q05) and (B) *M. tuberculosis* CsoR (PDB: 2HH7). The metal-binding domains are colored purple, the DNA-binding domains are colored teal, and the metal ions are orange spheres.

Typically, metalloregulatory transcription factors are homo-oligomeric DNA-binding proteins. Fig. 1 illustrates the typical structure of a class of copper regulatory proteins. Generally, metalloregulators consist of an N-terminal DNA-binding domain and a C-terminal metal-binding domain. Metal coordination drives conformational changes in the protein to activate or repress transcription of specific regulatory genes. Activator proteins, in the absence of metal stress, interact with DNA to prevent RNA polymerase from binding to the DNA. Upon binding to Cu(I) ions, the activator will undergo a conformational change that causes a distortion in the DNA to allow RNA polymerase to interact with the protein-DNA complex for transcription to occur. The copper efflux regulator CueR is one type of activator with a primarily α-helical structure protein with three distinct domains: the DNA binding domain at the N terminus consisting of α1 to α4, the dimerization helix at α5, and the metal binding domain at the turn between α5 and α6 (Fig. 1 *A*) (19). In CueR, Cu(I) coordinates via two conserved cysteine residues (C112 and C120), forming a high-affinity coordination site. CueR binds Cu(I) ions with a high affinity (dissociation constant K_D_ from 10^-16^ to 10^-21^ M) to promote the transcription of copper regulatory genes *cueO* and *copA* (19,20,21,22). CueO is a multicopper oxidase that oxidizes Cu(I) into the less toxic Cu(II), whereas CopA functions as a Cu(I) scavenger that transports copper ions into the bacterial periplasm (23,24).

## Electron spin resonance spectroscopy

Electron spin resonance (ESR) spectroscopy is a powerful technique for capturing the dynamics and conformational states of a wide variety of biological systems. The method is uniquely advantageous due to its selectivity for species with unpaired electrons, enabling precise characterization of paramagnetic centers and their surrounding environments. Most biological macromolecules are naturally ESR silent due to the absence of paramagnetic species, with metalloproteins being a notable exception.

### Site-directed spin labeling

To study a biomolecule by ESR spectroscopy, a stable moiety with an unpaired electron must be introduced into the system. To this end, Hubbell and co-workers pioneered the use of ESR-active spin labels, establishing the method of site-directed spin labeling (SDSL) (25,26). In SDSL, a paramagnetic spin label is introduced at a specific site in a macromolecule, typically through site-directed mutagenesis followed by a post-synthetic chemical modification (27). Alternatively, spin labels can also be incorporated directly during macromolecular synthesis, though this approach is limited to relatively small peptides or proteins (28,29,30). For larger proteins, spin labels can also be introduced genetically using noncanonical amino acids or other site-specific incorporation strategies (31,32).

Spin labels can be broadly classified into nitroxide radicals, paramagnetic metal complexes, and triarylmethyl radicals. Among these, nitroxide radicals are the most commonly used due to their stability, relatively small size, and high sensitivity to changes in the local environment (26,33,34,35). Nitroxides are typically attached covalently to proteins via a cysteine residue introduced at a specific site using thiol reactive groups, such as maleimides (36,37), iodoacetamide (38,39), or methanethiosulfonate groups (40,41,42,43). To enable specific labeling, native cysteines must be absent or inaccessible to the label. If native cysteines are present and not essential to structure or function, they are mutated to prevent nonspecific labeling, allowing for the site-specific introduction of a cysteine residue at the desired position (44,45).

One of the most commonly used and well-characterized nitroxide spin labels in SDSL is (1-oxyl-2,2,5,5-tetramethylpyrroline-3-methyl)methanethiosulfonate spin label (MTSSL). However, like many spin labels, MTSSL exhibits some conformational flexibility, which can lead to ambiguities in the interpretation of distances in terms of protein structures. In cases where cysteine-based labeling is not possible or a rigid label is desired, alternative labeling strategies are employed, which include labeling via histidine residues (46,47) or noncanonical amino acids (31,48,49,50).

While nitroxides are the focus of this review, cysteine-based approaches have also enabled the incorporation of other spin labels. Each of the unpaired electron species has its own unique advantages. For example, gadolinium(III) complexes are particularly useful for sensitive in-cell ESR applications, as they are relatively resistant to reduction under native cellular conditions (51,52,53). Further, trityl spin labels have narrow linewidths and slow relaxation times, making them excellent for structural measurements at higher temperatures (and even physiological temperatures) or for in-cell measurements (54,55,56,57,58,59,60,61). Copper(II) and manganese(II) spin labels with cysteine attachments have also been developed and have their own unique advantages (62,63,64). Spin labeling has also been applied to nucleic acids through post-synthetic chemical modification or incorporation of modified nucleotides during synthesis (65,66).

Currently, nitroxides remain the most widely used, as the spectral features of nitroxide labels are well studied, making them ideal for both continuous-wave and pulsed ESR measurements. This review exclusively uses nitroxides to measure the role of backbone conformations in metalloregulator function. Therefore, the following section provides a brief overview of the conceptual basis for these applications.

### Theory of continuous-wave ESR

In the presence of an external magnetic field, the spin of an unpaired electron interacts with the field and nearby electron and nuclear spins. The general spin Hamiltonian for an electron spin in a magnetic field can be written as

(1) $$\hat{H}={{\hat{H}}_{EZ}+{\hat{H}}_{NZ}+{\hat{H}}_{HF}=\beta }_{e}\vec{B}\cdot \overset{↔}{g}\cdot \hat{S}-\beta_{n}\sum_{i=1}^{k}\left(\vec{B}\cdot {\overset{↔}{g}}_{n,i}\cdot {\hat{I}}_{i}\right)+\hbar \sum_{i=1}^{k}\left(\hat{S}\cdot \overset{↔}{A_{i}}\cdot {\hat{I}}_{i}\right)\text{,}$$

where ${\hat{H}}_{EZ}$ is the electron Zeeman interaction, ${\hat{H}}_{NZ}$ is the nuclear Zeeman interaction, and ${\hat{H}}_{HF}$ is the hyperfine interaction, all expressed in energy units (Joules, J). In this equation, $\overset{↔}{g}$ and ${\overset{↔}{g}}_{n,i}$ are the g-tensors of the electron and nuclear spins, respectively (dimensionless), *β*_*e*_ and *β*_*n*_ are the Bohr and nuclear magnetons, respectively, with units of Joules per tesla (J/T), and $\vec{B}$ is the applied magnetic field vector (tesla, T). The hyperfine coupling tensors $\overset{↔}{A_{i}}$ are given in units of inverse seconds (s^−1^), and *ℏ* is the reduced Planck’s constant (J·s). We define the dimensionless spin angular momentum operators as $\hat{S}$ and ${\hat{I}}_{i}$ for the electron and nuclei, respectively.

The magnitude of the nuclear Zeeman interaction is much weaker than the electron Zeeman interaction due to the much smaller nuclear magnetic moment and can be often neglected for CW-ESR. Other interactions, including nuclear quadrupole coupling, zero-field splitting, and Heisenberg exchange interactions, are not considered here as they are not relevant to the present discussion (see reference (67) for complete discussion).

For nitroxide spin labels, the dominant interactions are the electron Zeeman and hyperfine coupling between the unpaired electron and the ^14^N nucleus (nuclear spin *I* = 1). The relevant spin Hamiltonian is therefore simplified to

(2) $$\hat{H}={{\hat{H}}_{EZ}+{\hat{H}}_{HF}=\beta }_{e}\vec{B}\cdot \overset{↔}{g}\cdot \hat{S}+\hbar \hat{S}\cdot \overset{↔}{A}\cdot \hat{I}$$

In typical magnetic fields of CW-ESR experiments, the electron Zeeman interaction is much larger than the hyperfine interaction. Under the high-field approximation and choosing the magnetic field to be applied along the z axis, the Hamiltonian can be rewritten as

(3) $$\hat{H}\approx \beta_{e}B_{0}g_{eff}\hat{S_{z}}+{\hbar A}_{eff}\hat{S_{z}}\hat{I_{z}}\text{,}$$

where *B*_0_ is the z-component of the applied magnetic field, and *g*_*eff*_ and *A*_*eff*_ are the projections of the g and hyperfine tensors onto the magnetic field axis and depend on the orientation of the magnetic field. For rhombic nitroxide systems, these are defined as follows (68):

(4) $$g_{eff}=\sqrt{{g_{xx}}^{2}\;\sin^{2}\;\theta \;\cos^{2}\;\phi +{g_{yy}}^{2}\;\sin^{2}\;\theta \;\sin^{2}\;\phi +{g_{zz}}^{2}\;\cos^{2}\;\theta }$$

(5) $$A_{eff}=\sqrt{{A_{xx}}^{2}\;\sin^{2}\;\theta \;\cos^{2}\;\phi +{A_{yy}}^{2}\;\sin^{2}\;\theta \;\sin^{2}\;\phi +{A_{zz}}^{2}\;\cos^{2}\;\theta }$$

Here, *g*_*xx*_, *g*_*yy*_, and *g*_*zz*_ are the principal axes of the g-tensor, and *A*_*xx*_, *A*_*yy*_, and *A*_*zz*_ are the principal axes of the hyperfine tensor. *θ* and *ϕ* are the polar and azimuthal angles, respectively, where *θ* is the angle between the magnetic field vector and the molecular zz-axis, and *ϕ* is the angle between the xx-axis and the projection of the magnetic field vector onto the xy plane. The expressions are given in the principal axis frame of the tensors, so the off-diagonal terms vanish (68,69).

When an external magnetic field is applied, the electron Zeeman interaction breaks the degeneracy of the electron spin energy levels, yielding two quantized energy levels for the spin states. The corresponding energies for the two spin states characterized by the quantum numbers $m_{S}=\pm \frac{1}{2}$ are given by the eigenvalues of $\hat{H}$:

(6) $$E=\beta_{e}B_{0}g_{eff}m_{s}+\hbar A_{eff}m_{s}m_{I}$$

**Figure 2:**
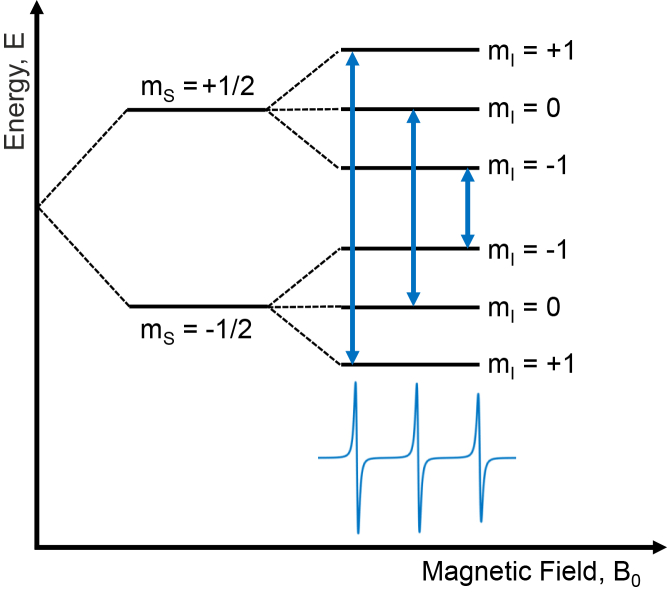
Energy level diagram for nitroxide. In the absence of a magnetic field, the energy levels are degenerate. When an external magnetic field is applied, the electron spin interacts with the field by the Zeeman interaction, resulting in an energy split. The energy levels are split further due to the hyperfine interaction between the electron and nitrogen nuclear spin. There are three allowed energy transitions, as indicated by the arrows. Each transition results in a peak in the CW-ESR spectrum.

The hyperfine interaction energy includes the nuclear spin angular momentum quantum number *m*_*I*_. Due to the hyperfine interaction, each electron spin state splits further into 2*I* +1 sublevels. In the case of nitroxide spin labels, the unpaired electron interacts with nitrogen (^14^N), which has a nuclear spin quantum number *I* = 1. There will be three sublevels, corresponding to nuclear spin states, *m*_*I*_ = +1, 0, −1. As a result, there are three allowed ESR transitions for nitroxide, under the selection rules Δ*m*_*S*_ = ±1 and Δ*m*_*I*_ = 0. Fig. 2 shows the electron spin energy levels for a nitroxide system. These transitions follow the resonance condition:

(7) $$\Delta E=h\nu =\beta_{e}B_{0}g_{eff}+\hbar A_{eff}m_{I}\text{,}$$

where *ν* is the microwave frequency. In CW-ESR, the magnetic field is swept while irradiating the sample at a fixed microwave frequency. Resonance occurs when the energy of the microwave radiation *hν* matches the energy difference between the spin states, inducing transitions between energy levels. In nitroxide-labeled systems, the transitions give rise to a characteristic three-line CW-ESR spectrum.

### Continuous-wave ESR for protein dynamics

**Figure 3:**
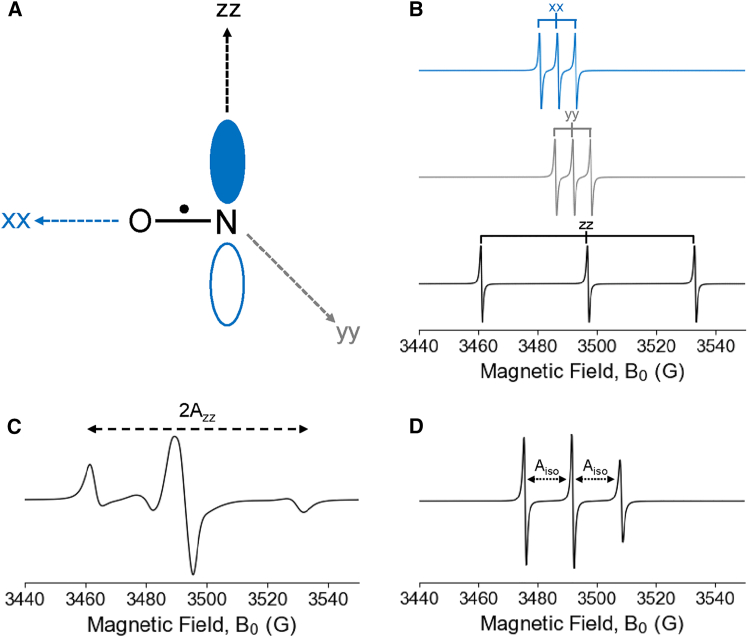
Anisotropy of nitroxide radical. (A) The orientation of the g and hyperfine tensors with respect to nitroxide is defined along three axes. The g_zz_ axis corresponds to the direction of the p_z_ orbital lobes on the nitrogen atom. (B) Illustration of the orientational effects of the g-values and hyperfine splitting. (C) The nitroxide spectrum at the rigid limit, where only the A_zz_ hyperfine splitting is resolved. (D) The nitroxide spectrum in the isotropic case, where the hyperfine splitting is reduced to the isotropic value, A_iso_.

Both the g and hyperfine tensors in the previous equations are dependent on the orientation of the nitroxide with respect to the magnetic field. Fig. 3 *A* shows a nitroxide radical in the molecular frame, which is defined by the principal axes of the g-tensor: *g*_*xx*_, *g*_*yy*_, and *g*_*zz*_. The *g*_*zz*_ axis is along the 2p_z_ orbital of the nitrogen, the *g*_*xx*_ axis is along the nitrogen-oxygen bond, and *g*_*yy*_ is orthogonal to both axes. Typical g-values for nitroxide radicals are approximately 2.008, 2.005, and 2.002 for *g*_*xx*_, *g*_*yy*_, and *g*_*zz*_, respectively. Similarly, the hyperfine components are approximately 12–13 MHz for *A*_*xx*_ and *A*_*yy*_, and 92–103 MHz for *A*_*zz*_, (70) though the hyperfine values will depend on the system and local electrostatics (71,72). A schematic illustration of the orientational dependence of the nitroxide spectrum is shown in Fig. 3 *B*. Here, the spectra were calculated for the special cases in which the magnetic field is directed along each of the three principal axes individually. Due to the difference in g-values and A-values, the resonant field positions and hyperfine splitting values differ for these orientations. The three spectra are not observed separately in experiment but instead indicate the limiting resonance positions of the anisotropic g and hyperfine tensors. In reality, the observed spectrum of the nitroxide spin label reflects an orientational average over all possible molecular orientations.

In one case, the nitroxide spin label is in the rigid limit, such as in frozen solutions or solid-state samples. Here, the anisotropy of the g and hyperfine tensors becomes observable, and the CW-ESR spectrum becomes dependent on the orientation of the molecule with respect to the magnetic field. The combined orientational dependence of the g and hyperfine tensors produce the rigid-limit (powder) ESR spectrum, as shown in Fig. 3 *C*, which is the summed contribution of all possible nitroxide orientations with respect to the magnetic field. In this case, the outer edges of the spectrum are defined by the A_zz_ component, while the other orientational components contribute to the interior of the spectrum and are not resolved (70).

In the opposite limit, in a low-viscosity solution, the spin label reorients rapidly relative to the ESR timescale (i.e., at rotational rates of >10^10^ s^−1^ at ∼9.8 GHz), and thus, the anisotropy in the g and hyperfine tensors will average out. Under these conditions, the system can be approximated as isotropic, and the spin Hamiltonian is represented as

(8) $$\hat{H}=\beta_{e}B_{0}g_{iso}{\hat{S}}_{z}+A_{iso}{\hat{S}}_{z}{\hat{I}}_{z}\text{,}$$

where the isotropic tensor components are defined as

(9) $$g_{iso}=\frac{1}{3}\left(g_{xx}+g_{yy}+g_{zz}\right)$$

(10) $$A_{iso}=\frac{1}{3}\left(A_{xx}+A_{yy}+A_{zz}\right)$$

In this isotropic limit, the anisotropy in the g and hyperfine tensors completely average out, resulting in three sharp lines positioned and split by the average tensor values of *g*_*iso*_ and *A*_*iso*_ of about 2.005 and 16 G, respectively. Fig. 3 *D* shows the isotropic CW-ESR spectrum.

**Figure 4:**
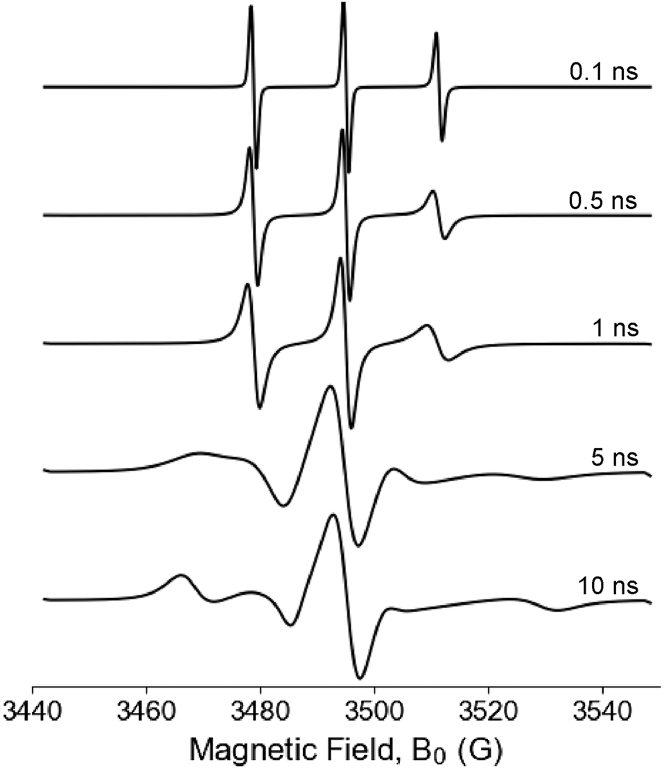
CW-ESR spectra of a nitroxide spin at different rotational correlation times (τ_corr_) simulated by EasySpin.

Fig. 3 *C*, *D* represent the two extremes of the orientational dependence of the CW-ESR spectrum: completely rigid and fully isotropic. However, a nitroxide attached to a macromolecule will fall between these two extremes where the g and hyperfine tensors are partially averaged. As a result, the CW-ESR lineshape becomes sensitive to the motional dynamics of the system. Fig. 4 illustrates multiple simulated nitroxide spectra at different rotational correlation times (τ_corr_), which reflect varying degrees of molecular mobility. The simulations were performed using EasySpin (73). These simulations demonstrate that the CW-ESR spectral lineshape is sensitive to changes in rotational rates, making nitroxide spin labels powerful probes of site-specific dynamics. At X-band frequencies (∼9.8 GHz), CW-ESR is sensitive to motions with τ_corr_ on timescales between 0.1 and 100 ns (74). Within this time window, multiple types of molecular reorientation can impact the ESR spectral lineshape, including global tumbling of the macromolecule (i.e., Brownian rotational diffusion), backbone fluctuations at the labeling site, and local side-chain motions of the label.

The influence of molecular motion on the CW-ESR lineshape depends on whether the motion is fast or slow compared with the electron Larmor precession frequency, which depends on the magnetic field strength. For example, at the magnetic fields typically used in nitroxide CW-ESR (0.3–0.4 T, X-band), global rotational tumbling of large proteins is typically too slow to contribute to the CW-ESR. However, for smaller proteins (less than 50 kDa), global rotational motion can fall within the ESR-sensitive timescale and impact the lineshape. This global motion can mask more informative spectral features that arise from local protein dynamics, such as backbone fluctuations. To suppress the contribution of global tumbling to the spectrum, the viscosity of the solution is increased by adding agents such as Ficoll. This additive slows global tumbling without impacting the internal motion of the spin label (75,76). By minimizing tumbling, the CW-ESR spectrum becomes more sensitive to site-specific dynamics, particularly backbone fluctuations, which are often the most informative for understanding how local protein dynamics relate to biological function.

**Figure 5:**
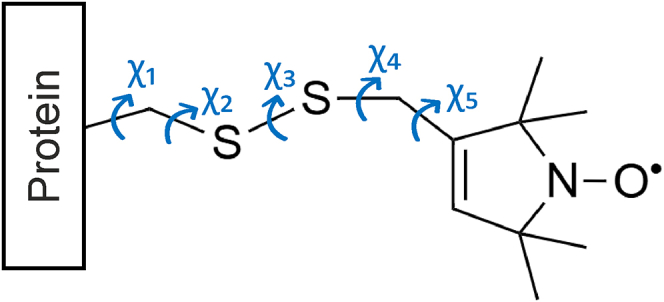
Structure of the R1 side chain formed by the reaction of MTSSL with a cysteine residue. Dihedral angles χ_1_-χ_5_ are marked in blue.

However, interpreting the influence of backbone dynamics on the CW-ESR spectrum is complicated by the local motions of the spin-labeled side chain. For MTSSL, the dominant side-chain motion arises from torsional oscillations about the five rotatable bonds in the side chain (designated R1). Fig. 5 shows the relevant dihedral angles. These torsions allow the nitroxide ring to adopt multiple rotamers and potentially interact with neighboring amino acid residues.

Nonetheless, studies have shown that at solvent-exposed α-helical sites, side-chain interactions are minimal, and R1 motion is highly conserved. At these sites, X-ray crystallography (77,78,79,80) and computational modeling (81,82,83) have revealed preferred R1 rotamers, stabilized by hydrogen bonding interactions that restrict rotation about the bonds with dihedral angles χ_1_ and χ_2_. Additionally, the χ_3_ angle has inherently slow oscillations, and the remaining χ_4_ and χ_5_ dihedrals dominate the motion of the nitroxide ring. This led to the development of the χ_4_/χ_5_ model, which describes the motional behavior of R1 at solvent-exposed α-helical sites.

R1 motions were shown to be conserved along the surface-exposed face of an α helix, so variations in CW-ESR lineshape were attributed primarily to differences in backbone dynamics (77). This interpretation was further supported by spectral fitting using the microscopic order-macroscopic disorder (MOMD) model, which incorporates spatial ordering potentials and allows for extraction of rotational correlation times and order parameters for the labeled site (84,85). Here, the order parameter quantifies the degree of angular restriction of the spin label relative to the protein backbone, where a value of 0 corresponds to isotropic motion, and 1 corresponds to perfect alignment (73,84). This order parameter describes spatial ordering and should not be confused with the squared order parameter used in NMR relaxation. For rigid, surface-exposed α helices, these parameters are conserved, allowing for a quantitative assessment of site-specific backbone fluctuations (75).

Because the spin label is connected to the protein via a flexible side chain, its motion reflects both the internal flexibility of the R1 side chain and the structural dynamics of the local protein environment. As a result, variations in the CW-ESR lineshape can be attributed to differences in local protein dynamics. This makes the motion of the spin label a sensitive and reliable reporter of site-specific backbone motion. In contrast, similar analyses of R1-labeled β sheet structures showed greater variability in rotamers, indicating less predictable side-chain motions (86,87,88). Therefore, it is more difficult to extract relevant protein dynamics information in β sheets.

Additional methods of distinguishing side-chain rotations from backbone dynamics include pressure-dependent CW-ESR (89,90), two-dimensional pulsed ESR experiments (72,91,92,93), and high frequency ESR (94,95). Overall, information on protein backbone dynamics can be extracted from the CW-ESR lineshapes of surface-exposed α-helical structures since the side-chain motions are conserved in these structures.

Having established the experimental framework for detecting dynamics, it is important to consider why protein dynamics matter. Protein functions, such as enzymatic catalysis, molecular recognition, and allosteric regulation, often depend on a wide range of motions that occur across a range of timescales. Understanding these dynamics is essential to gaining a complete picture of a protein structure-function relationship.

## Copper efflux regulator, CueR

CueR regulation is conserved across multiple bacterial systems, including the clinically relevant pathogen *Pseudomonas aeruginosa*. The *E. coli* and *P. aeruginosa* CueR proteins share a high degree of sequence similarity (64%) and identity (47%), but they differ in their response to copper. For example, the binding affinity to Cu(I) is different in each system, where *E. coli* CueR responds to a lower concentration of Cu(I) in the cell (dissociation constant, K_D_ ∼ 10^-21^ M) than *P. aeruginosa* (K_D_ ∼ 10^-16^ M) (19,20,21,22). In addition, CueR facilitates the expression of different copper tolerance genes in each bacterium. In *E. coli*, two genes are expressed, a Cu(I) ATPase and a multicopper oxidase (23,24), while in *P. aeruginosa* the same Cu(I) ATPase is expressed in addition to two Cu(I) chaperones and a multidrug efflux pump (20,96,97). Although the link between the efflux pump and copper homeostasis remains unclear, its role in removing antibiotics and other foreign substrates may contribute to the multidrug resistance of *P. aeruginosa*. Despite these differences, both CueR homologs activate the expression of remedial proteins by interacting with DNA upon Cu(I) stress. However, the details of the conformational changes that drive the mechanism remain poorly understood.

To address these questions, a range of structural and biophysical approaches have been used. While the structure of *P. aeruginosa* CueR has not yet been resolved by X-ray crystallography, the structure of *E. coli* CueR has been resolved (Fig. 1 *A*). Additional X-ray structures of the *E. coli* CueR-DNA complex in both metal-free (PDB: 4WLS) and metal-bound (PDB: 4WLW) states show that DNA can interact even in the absence of Cu(I) (98). However, in the presence of Cu(I), the protein induces a bend in the DNA to facilitate transcription. These findings are supported by a more recent cryo-EM structure showing the same distorted DNA conformation in the presence of Cu(I) and RNA polymerase (99,100).

**Figure 6:**
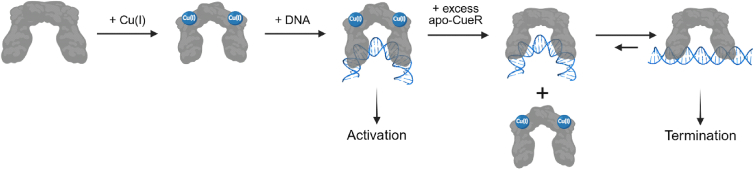
Cartoon representation of the CueR transcription regulation mechanism based on ESR measurements. Under Cu(I) stress, the CueR dimer will coordinate Cu(I), and the DNA binding domains will come closer to each other. Holo-CueR will interact with promoter DNA and induce a bend in the DNA, allowing RNA polymerase to coordinate to the complex and activate transcription. After Cu(I) homeostasis is restored, excess apo-CueR will directly substitute holo-CueR in the complex. The complex will shift into the more thermodynamically favorable linear DNA to terminate transcription. Figure is adapted from (103).

To better understand mechanistic details of CueR, solution-state conformational changes were measured by ESR spectroscopy coupled with SDSL. Double electron-electron resonance (DEER) spectroscopy was utilized to measure the conformational changes that occurred in CueR upon DNA coordination. Distinct DEER distance distributions were observed for apo (metal-free), holo (metal-bound), repressed (metal-free CueR-DNA complex), and active (metal-bound CueR-DNA complex) states of *E. coli* CueR (101,102). Additionally, distance measurements on the spin-labeled promoter DNA sequence were collected in different functional states (103). Based on the combined DEER distances, a mechanism for transcription activation was proposed, as illustrated in Fig. 6. After Cu(I) coordination to each monomer of CueR, the DNA-binding domains of the dimeric structure move closer together, suggesting a squeezing motion facilitates the protein-DNA interaction (102). Moreover, upon the interaction with holo-CueR, the distance between the spin-labeled sites on the DNA became smaller. The bending of the DNA is essential for transcription, as the distortion allows for the sites needed for RNA polymerase interaction to be in the correct orientation for binding (100,104,105). Thus, transcription of proteins required for metal remediation is activated. Notably, we found that the distortion of the DNA also occurred in the presence of excess apo-CueR, a finding that was surprising in light of the crystal structure of the CueR-DNA complex (103). On the other hand, the finding that apo-CueR can also induce DNA distortion similarly to holo-CueR suggests that DNA distortion is not entirely dependent on Cu(I) binding. More importantly, the data suggest that upon amelioration of Cu(I), excess CueR can easily substitute holo-CueR on the DNA. This substitution is facile since apo-CueR is able to bind to bent DNA just as holo-CueR can. The complex can then transition into a state where the DNA becomes linear to terminate transcription of Cu(I) remedial proteins. This observation provides a structural context for a direct substitution mechanism of transcription termination that was proposed earlier (106). Overall, the DEER results demonstrate that CueR transitions through multiple, Cu(I)-dependent conformational states in solution, providing mechanistic insight into its role as a transcriptional activator.

## CueR dynamics

The DEER experiments provided clear evidence of conformational changes in both protein and DNA. More recently, comparative continuous-wave (CW) ESR measurements on CueR from two bacterial species were performed to monitor domain-specific changes in protein dynamics as a function of Cu(I) and DNA binding (107).

**Figure 7:**
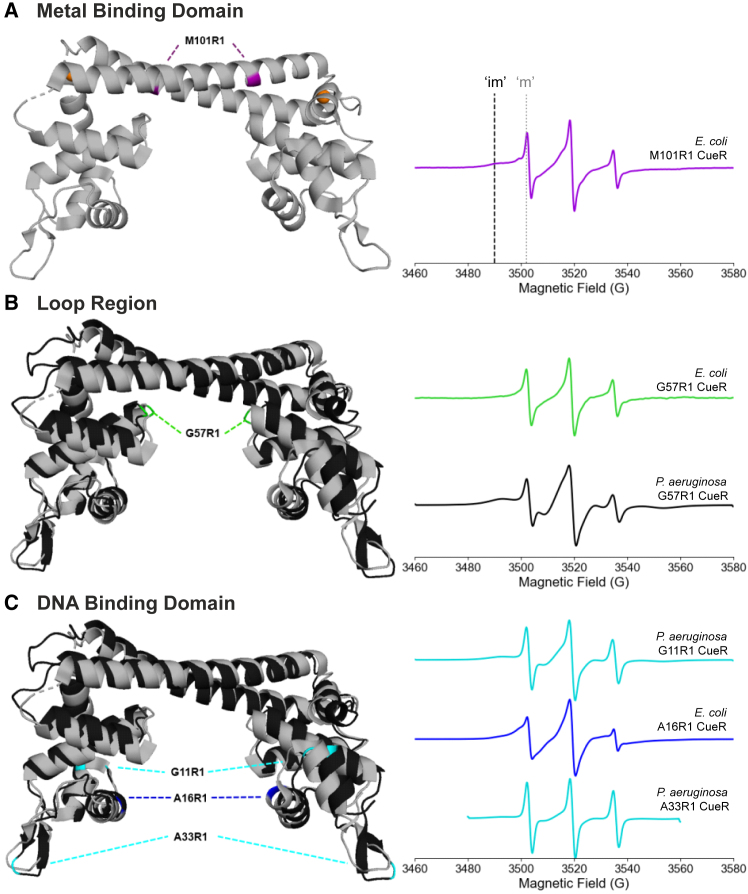
Site-specific dynamics of CueR. (A) X-ray crystal structure of *E. coli* CueR (PDB: 1Q05) (gray) with the M101R1 site colored purple. The orange spheres represent Cu(I) ions. On the right is the CW-ESR spectrum of the M101R1 *E. coli* CueR mutant in the apo state. The dashed black line indicates the immobile (“im”) component of the low-field line, and the dotted gray line indicates the mobile (“m”) component of the low-field line. (B) AlphaFold3-predicted structure of *P. aeruginosa* CueR (black) overlaid on the X-ray crystal structure of *E. coli* CueR (gray) with the G57R1 site colored green. The right plot shows the CW-ESR spectra for each protein in the apo state, green corresponds to *E. coli* CueR, and black to *P. aerguinosa* CueR. (C) Overlaid structures with the spin-labeled sites indicated for *E. coli* CueR are in dark blue (A16R1) and *P. aeruginosa* CueR in cyan (G11R1 [upper spectrum] and A33R1 [lower spectrum]). The right side of the figure shows the CW-ESR spectra for each mutant in the apo state. Figure is adapted from (107).

Room temperature CW-ESR spectroscopy was used on *E. coli* and *P. aeruginosa* CueR to determine the relationship between dynamical changes and function. Nitroxide spin labeling was employed at three different sites within the proteins. In both species, CueR has four native cysteine residues: C112 and C120 that function in the Cu(I) binding site and C129 and C130 at the C terminus. Spin labeling at the metal binding site was avoided by performing the reaction in the presence of Cu(I). In addition, C129 and C130 were mutated to alanine to prevent MTSSL labeling at these sites (107). In *E. coli* CueR, spin labels were introduced at three sites: M101R1 near the metal-binding domain, G57R1 in the loop between the metal-binding and DNA-binding domains, and A16R1 near the DNA-binding domain. Similarly, three positions were selected for labeling in *P. aeruginosa* CueR. G11R1 and A33R1 are located near the DNA-binding domain, and G57R1 is in the loop region between the two functional domains. The G57R1 mutant is used as a direct comparison between the two species. Fig. 7 illustrates these positions in each domain of the protein. Fig. 7 *B*, *C* show the structural alignment of the two protein species, with the crystal structure of *E. coli* CueR shown in gray and the AlphaFold-predicted structure of *P. aeruginosa* CueR shown in black.

Fig. 7 shows the room temperature CW-ESR spectra for the mutants in the apo state. While each spectrum has three characteristic peaks, there are distinct differences in the lineshapes between mutants. There are both broad and narrow components to each line in the spectra, as indicated by the vertical lines in Fig. 7 *A*. Such two-component spectra are commonly observed for spin-labeled proteins due to the spin label or protein populating two distinct motional states that interconvert slowly on the ESR timescale (18,33,34,88,108,109). The relative populations of these two components can change in response to changes in dynamics of the protein. In comparing M101R1 *E. coli* CueR to G57R1, for example, the broad component is more prominent in M101R1, suggesting restricted dynamics due to the location in the α helix. The dominant narrow component in G57R1 suggests faster dynamics at the site, which is expected as it is on a loop between two helices. In *P. aeruginosa* CueR, the spectrum of G57R1 is much broader than the other two mutants, indicative of slower dynamics at this loop region. The spectra of G11R1 and A33R1 have narrower lines, suggesting faster dynamics near the DNA-binding domain. These differences show that nitroxide room temperature CW-ESR spectra meaningfully measure site-specific dynamics in the CueR protein, such that changes in the lineshape upon addition of Cu(I) or DNA can be attributed to changes in the dynamics (107).

**Figure 8:**
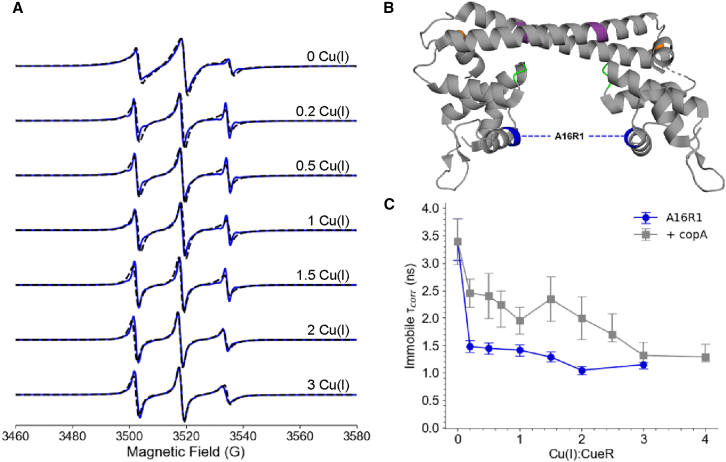
Dynamics of the DNA binding domain of *E. coli* CueR. (A) RT CW-ESR spectra (solid blue) for A16R1 *E. coli* CueR in the absence of DNA as a function of Cu(I) concentration per CueR monomer. The dashed black lines are the fits obtained from EasySpin simulations using the chili program. (B) Cartoon representation of the A16R1 mutant of *E. coli* CueR (PDB: 1Q05). (C) Rotational correlation time of the immobile component (τ_corr_) as a function of Cu(I):CueR ratio in the absence (blue circles) and presence of copA DNA (gray squares). Error bars represent 95% confidence intervals in the fits from EasySpin. Figure is adapted from (107).

To analyze the changes in site-specific dynamics, the CW-ESR spectra were recorded as a function of Cu(I) in both the protein-only and protein-DNA complexes. As an illustrative example, the spectra for the A16R1 mutant of *E. coli* CueR are shown in Fig. 8. The lineshape changes were quantified by simulating the spectra using the MOMD model implemented in EasySpin (73,84,85). The MOMD model relies on the spatial ordering potentials for the motion of the nitroxide spin label. In fitting CW-ESR spectra with the model, the rotational correlation time (τ_corr_) and order parameters of the motion of the spin label are systematically varied until a best fit is reached. An increase in site-specific dynamics results in a decrease in order parameter or increase in τ_corr_ (107). The correlation time of the protein side chain, detected in these studies, is on the order of a few ns, which is typically lower than the overall tumbling rate of the entire protein (110).

In the simulation of the A16R1 mutant of *E. coli* CueR, only the τ_corr_ of the immobile component was varied, as the inclusion of an order parameter was not needed to achieve a good fit. Fig. 8 *A* shows the CW-ESR spectra in the absence of DNA as a function of Cu(I). The lineshape generally became more narrow as Cu(I) was added. Such narrowing indicates faster motion with the addition of Cu(I). Fig. 8 *C* shows the decrease in the τ_corr_ as soon as even 0.2 equivalents of Cu(I) is added, indicating an increase in dynamics at the spin-labeled site. In these experiments, the labeled protein is present at 50 μM. Given the extremely high affinity of CueR for Cu(I), even sub-stoichiometric additions of Cu(I) will be effectively completely bound by the protein. Under these conditions, the observed changes in the CW-ESR lineshape with increasing Cu(I) ions arise from shifts in the dynamical states of the protein. At higher Cu(I) concentrations, the dynamics continue to increase. In the presence of copA DNA, τ_corr_ decreases with increasing Cu(I), as shown in Fig. 8 *C*, indicating that Cu(I) consistently promotes increased dynamics near the DNA-binding domain. However, in the absence of Cu(I), the dynamics at the DNA-binding domain are the same with and without DNA. This highlights that Cu(I), not DNA binding alone, drives the observed change in dynamics. This observation is especially notable, as the DNA-binding domain is more than 27 Å from the metal binding site, strongly supporting an allosteric mechanism by which Cu(I) binding influences DNA recognition. These fluctuations at the DNA binding domain likely facilitate the search and recognition of the copA DNA sequence for transcription regulation (107).

Conversely, the metal-binding domain exhibits decreased dynamics upon Cu(I) addition of one to two equivalents per CueR monomer, consistent with a tight interaction between CueR and the metal ion. Notably, a further decrease in dynamics beyond two equivalents suggests a second Cu(I) binding site per monomer (107). Previous work has also proposed the existence of a second Cu(I) binding site (101,102,111). In Fig. 8 *C*, the occupancy of the second binding site and its allosteric effects are represented as a smooth change in the correlation time rather than a sharp distinction. Together, the CW-ESR experiments and simulations in this work demonstrate that Cu(I) binding to CueR induces domain-specific changes in dynamics, providing strong evidence of an allosteric mechanism of action.

Unlike *E. coli* CueR, however, the dynamics of the DNA-binding domain in *P. aeruginosa* CueR appeared to be strongly influenced by DNA and, in particular, the DNA sequence itself. In the presence of mexPQ-opmE DNA, fast dynamics were observed even at low Cu(I) concentrations. With copZ2 DNA, the dynamics increased gradually with Cu(I) addition, eventually reaching similar τ_corr_ values as those seen with mexPQ-opmE DNA. The difference in dynamics may be explained by additional biochemical data suggesting that *P. aeruginosa* CueR has a higher affinity for copZ2 than for mexPQ-opmE DNA in the absence of Cu(I) (112). These results highlight DNA-dependent changes in local dynamics in *P. aeruginosa* CueR that is absent in *E. coli* CueR, where DNA binding did not significantly impact dynamics.

**Figure 9:**
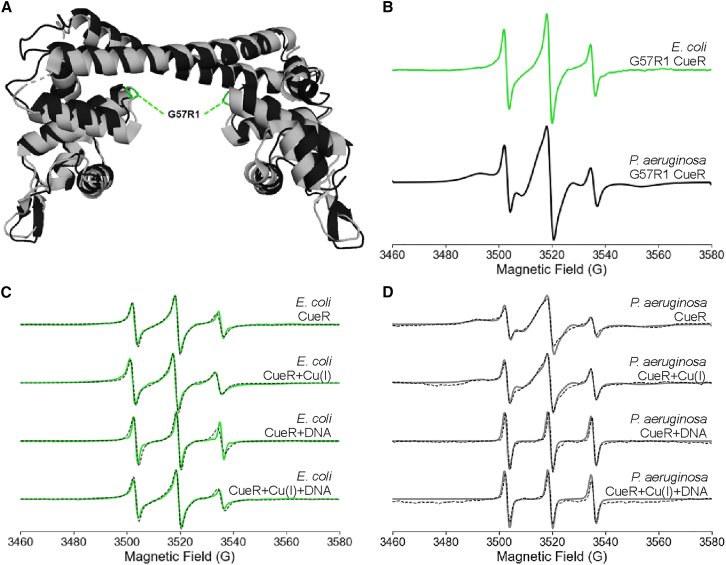
Comparison of site-specific dynamics in CueR proteins of different bacteria. (A) AlphaFold3-predicted structure of *P. aeruginosa* CueR (black) overlaid on the X-ray crystal structure of *E. coli* CueR (gray) with the G57R1 site colored green. (B) Room temperature CW-ESR spectra for the mutants in the apo state (*E. coli*, green, *top*) (*P. aeruginosa*, black, *bottom*). (C) G57R1 *E. coli* CueR (green) and (D) G57R1 *P. aeruginosa* CueR (gray) in four different functional states. The dashed black lines are the simulated fits from EasySpin. Figure is adapted from (112).

As a direct comparison between the two CueR species, the dynamics at the G57R1 site, a loop between α3 and α4, were measured in each protein. Fig. 9 *A* illustrates the site on both CueR structures. Fig. 9 *B* shows a narrower spectral lineshape for *E. coli* compared with *P. aeruginosa* in the apo state, indicating faster dynamics in *E. coli* CueR at this loop region. Interestingly though, the predicted structure suggests no major differences in backbone conformation at this site. One explanation for the difference in dynamics is the presence of π-π stacking between α3 and α4 helices in *P. aeruginosa* CueR, which is missing in *E. coli* CueR. The stacking interactions likely stabilize the loop, restricting motion and resulting in the broader spectral lines. Upon the addition of Cu(I) and/or DNA, the dynamics at G57R1 remain largely constant in *E. coli* CueR, as shown by the spectra in Fig. 9 *C*. On the other hand, Fig. 9 *D* shows the dynamics are greatly influenced by the addition of DNA and Cu(I) in *P. aeruginosa* CueR. The spectrum narrows slightly upon the addition of Cu(I) and becomes even more narrow with DNA, indicating increased dynamics at the G57R1 site. The increase in dynamics by the addition of DNA further supports the influence of DNA on the *P. aeruginosa* CueR mechanism (112).

Overall, these comparative measurements reveal distinct Cu(I) response mechanisms in *E. coli* and *P. aeruginosa* CueR. *E. coli* CueR exhibits an immediate increase in the dynamics at the DNA-binding domain, at even low concentrations of the metal ion, reflecting a rapid allosteric activation mechanism. In contrast, *P. aeruginosa* CueR shows dynamics that are more tightly coupled to DNA binding and require higher Cu(I) levels for activation in the absence of DNA.

One possible explanation is that *P. aeruginosa* has multiple Cu(I) defense mechanisms that are not present in *E. coli*, including metallothionein proteins and cytoplasmic copper chaperones like copZ2, that regulate intracellular copper (21,113). CopZ2 is highly upregulated in *P. aeruginosa* upon exposure to Cu(I) and is suggested to function as a Cu(I) storage pool, enabling a rapid cellular response to elevated copper levels (21). As a result, CueR in *P. aeruginosa* may not need to respond as quickly to Cu(I) stress as *E. coli* CueR.

These findings highlight the importance of measuring protein dynamics to elucidate differences between structurally similar proteins, which may not be apparent from large-scale conformational changes alone. Despite the high sequence similarity between *E. coli* CueR and *P. aeruginosa* CueR, the site-specific dynamics of each protein evolved differently, resulting in distinct regulatory behaviors. While static structures can reveal conformational states and potential binding sites, dynamics studies provide complementary information by revealing how homologous proteins from different organisms can respond differently to metal ion stress, even when their overall structures are similar.

## Copper-sensitive operon repressor, CsoR

The copper-sensitive operon repressor CsoR was originally identified in *Mycobacterium tuberculosis* as a member of a major Cu(I) sensing family of bacterial metalloregulators. CsoR represses transcription of both its own gene and a Cu(I) efflux ATPase (CtpV) by binding to DNA in the apo state. Under Cu(I) stress, the protein coordinates Cu(I) with high affinity (K_D_ ≈ 10^−18^ M) and dissociates from DNA, allowing transcription of genes involved in copper detoxification (114).

Structural studies of CsoR have revealed conflicting models for its oligomeric state. The crystal structure of *M. tuberculosis* CsoR was resolved as an α-helical dimer (114), but subsequent studies of different CsoR species, *Bacillus subtilis*, *Thermus thermophilus*, and *Streptomyces lividans*, form tetramers arranged as dimers of dimers (115,116,117). In the case of *B. subtilis*, it has been proposed that two CsoR tetramers interact with a single operator DNA with 2:1 binding stoichiometry (115). However, CsoR lacks the canonical DNA-binding domain, unlike in CueR and other metalloregulators (118), and no high-resolution structure currently supports this 2:1 interaction model (119). Thus, it is unclear exactly how CsoR interacts with DNA in the apo state.

The elucidation of the oligomeric state of CsoR in solution, particularly in the presence of DNA, can provide more information on the regulation of Cu(I) toxicity in *M. tuberculosis* and similar bacterial species. While DEER measurements have recently been applied to probe conformational changes upon DNA binding, they revealed only minor changes (120). More recently, a detailed analysis of the oligomeric state of *M. tuberculosis* CsoR was performed using room temperature CW-ESR spectroscopy, pulsed electron paramagnetic resonance, and size exclusion chromatography (121).

### CsoR oligomerization

**Figure 10:**
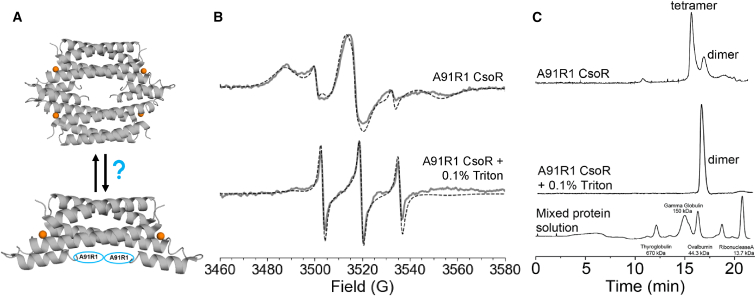
Oligomeric analysis of *M. tuberculosis* CsoR. (A) Nitroxide spin-labeled site in the crystal structure of *M. tuberculosis* CsoR (PDB: 2HH7) on the dimer (*bottom*). The arrows represent a potential equilibrium between the dimeric and tetrameric forms of CsoR. (B) Room temperature CW-ESR spectra of A91R1 CsoR in the absence (*top*) and presence of 0.1% Triton X-100 (*bottom*). The corresponding EasySpin simulations are shown as dashed black lines. (C) HPLC-SEC data of A91R1 CsoR in the absence (*top*) and presence of 0.1% Triton X-100 (*middle*). A mixed protein standard solution is provided for comparison of molecular weights (*bottom*). Figure is adapted from (121).

In this study, room temperature CW-ESR spectroscopy was performed to determine the changes in dynamics upon oligomerization. The A91C mutant of *M. tuberculosis* CsoR was spin-labeled with nitroxide. The A91R1 site is located in the C terminus of the protein near the oligomerization interface, based on the tetrameric crystal structure of CsoR. Fig. 10 *A* shows the placement of the A91R1 sites as ovals, as the C termini were not resolved in the crystal structure (121). Both the tetrameric and dimeric forms of CsoR are presented in Fig. 10.

Fig. 10 *B* shows the room temperature CW-ESR spectra of A91R1 CsoR in the absence and presence of 0.1% Triton X-100, a nonionic surfactant that can disrupt protein oligomerization. Each spectrum contains two components: a broad (immobile) and a narrow (mobile) component. In the absence of Triton X-100, the spectrum has a significantly broad lineshape, indicative of slow dynamics at the A91R1 site. Upon addition of Triton, the spectrum contains more of the narrow component, indicating increased dynamics due to disruption of oligomerization. Two-component EasySpin simulations were used to estimate the changes in populations of each component. In the simulations, only the weights of the components were varied while keeping dynamic parameters fixed, though with a slight expense on the quality of the fit. Without Triton, only 20% of the spectrum consisted of the mobile component, while with Triton, the fraction increased to 80%. The increase in the mobile component is consistent with reduced steric hindrance or dipolar interactions upon dissociation of the tetrameric assembly, and the mobile component is therefore attributed to the dimeric form of CsoR. To validate this assignment, high-performance liquid chromatography (HPLC) size-exclusion chromatography (SEC) was employed, as shown in Fig. 10 *C*. Without Triton, two elution peaks corresponding to 24 kDa and 48 kDa were observed, consistent with coexisting dimeric and tetrameric assemblies, respectively, with a higher population of tetramer. In the presence of Triton, a single chromatographic peak was present at the longer retention time, suggesting a completely dimeric protein (121).

**Figure 11:**
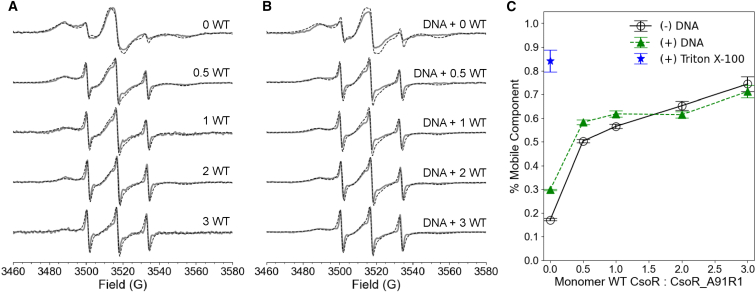
Site-specific dynamics of *M. tuberculosis* CsoR. (A) Room temperature CW-ESR spectra of A91R1 CsoR as a function of WT CsoR in the absence of DNA and (B) in the presence of DNA. The simulated spectra are shown in dashed black lines. (C) The fraction of mobile component in the two-component simulations as a function of WT CsoR for the protein only (open black circles), in the presence of DNA (green triangles), and in the presence of Triton X-100 (blue star). The error bars were calculated as 95% confidence intervals in EasySpin. Figure is adapted from (121).

To further analyze the oligomerization state of CsoR, the A91R1 mutant was titrated with unlabeled, wild-type (WT) CsoR while maintaining constant total protein concentration. Fig. 11 *A*, *B* show room temperature CW-ESR spectra as a function of the ratio of WT CsoR to A91R1 CsoR in the absence and presence of DNA, respectively. For example, “2 WT” corresponds to two molar equivalents of WT CsoR per one equivalent of A91R1 CsoR. Fig. 11 *C* shows that prior to the addition of WT CsoR, the mobile component initially comprised 20% and 30% of the spectra in the absence and presence of DNA, respectively (121).

**Figure 12:**
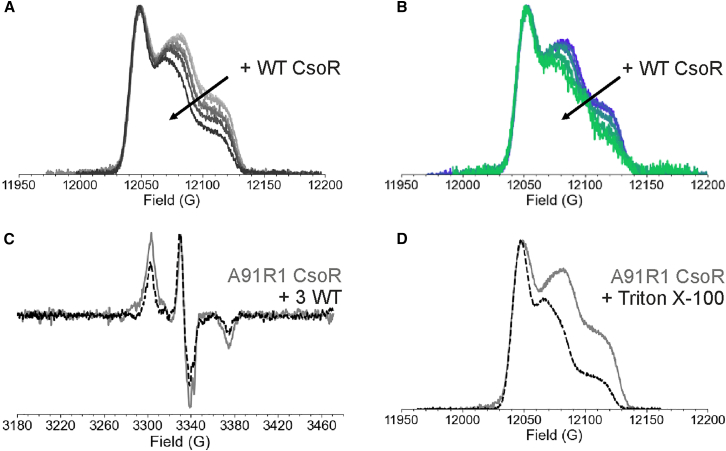
ESR analysis of CsoR oligomeric state. (A) Field-swept electron spin echo (FS-ESE) spectra at Q-band for A91R1 CsoR with increasing amounts of WT CsoR (light to dark) for CsoR without DNA and (B) with DNA (purple to green). (C) X-band CW-ESR spectra at 80 K of A91R1 CsoR (gray) and with 3 WT CsoR (dashed black). (D) FS-ESE spectra of A91R1 CsoR (gray) and with Triton X-100 (dashed black). Figure is adapted from (121).

As WT CsoR was added, the fraction of mobile component in the CW-ESR spectra increased, regardless of the absence or presence of DNA. While the total concentration of dimer and tetramer should remain unchanged, the introduction of non-spin-labeled protein reduces the number of spins per oligomer, leading to spin dilution. In tetramers, dipolar interactions between nearby unpaired electrons can cause line broadening in the ESR spectra. The more spins that are close together, the greater is the dipolar broadening (70). This interpretation was further supported by pulsed ESR field-swept electron spin echo spectra, in Fig. 12 *A*, *B*, which show a decrease in dipolar broadening as a function of WT CsoR. Further evidence comes from an 80 K CW-ESR spectrum, where the A91R1 CsoR tetramer shows stronger dipolar broadening compared with a sample with three equivalents of WT CsoR, as shown in Fig. 12 *C*. Finally, the field-swept spectrum in the presence of Triton X-100, shown in Fig. 12 *D*, also displays a weaker dipolar interaction compared with the native, tetrameric state of A91R1 CsoR, consistent with dissociation into the dimeric form (121).

Together, the CW-ESR spectra, HPLC-SEC results, and the pulsed ESR experiments demonstrate that A91R1 CsoR exists in a dynamic equilibrium between dimer and tetramer in solution. The equilibrium can shift toward the dimeric population by the addition of Triton X-100 or exchange of spin-labeled protein with WT protein. While DNA binding does not significantly alter the oligomeric state of CsoR, additional experiments suggest that the rate of subunit exchange within the oligomer is moderately affected by the presence of DNA. Particularly, the replacement of spin-labeled protein with WT protein appears to occur more readily and is less restricted in the presence of DNA. This is consistent with the slightly higher mobile component population in the presence of DNA in the CW-ESR spectra (121). One possible explanation is that DNA may position itself between two CsoR dimers, allowing for subunit exchange by partially disrupting contacts between the tetrameric form. This finding may be relevant to the CsoR mechanism, as the exchange between subunits could contribute to the dissociation of the protein from the DNA upon Cu(I) binding, enabling transcription initiation. These results highlight the unique mechanism of CsoR and the power of ESR spectroscopy in uncovering solution-state behavior inaccessible by crystallography.

## From copper metalloregulators to metalloproteins: Broader applications of ESR

The detailed studies of CueR and CsoR highlight the mechanistic and dynamic insights that ESR spectroscopy can provide. Nevertheless, these two systems represent only a small subset of the copper regulatory proteins that have been investigated by ESR spectroscopy. For example, CopY is a metalloregulator that activates transcription in the presence of Cu(I) ions and represses transcription in the presence of Zn(II) ions. The effects of these ions on the conformational and dynamical changes of the protein have been investigated by ESR spectroscopy (122). In addition, the gating mechanism of human copper transporter hCtr1 was elucidated by probing conformational changes using SDSL and pulsed ESR spectroscopy (123).

More broadly, ESR spectroscopy has been widely applied to a broad range of metalloregulators and metalloproteins. In many cases, these studies make use of the ability of ESR to directly probe paramagnetic metal ions without the need for spin labels. For instance, the reduction-oxidation cycling of transcriptional regulator SoxR in response to exogenous nitric oxide was characterized by CW-ESR (124). Likewise, CW-ESR was used to quantify nanomolar to micromolar binding affinities of Mn(II) to manganese transport regulator, and endogenous Fe(III) signals have been used to characterize ferritin iron clusters directly in cells (125,126). These examples illustrate the diversity of metal-responsive proteins studied by ESR spectroscopy, and comprehensive overviews of ESR applications to metalloenzymes and metalloproteins are available elsewhere (127,128).

## Conclusion

CueR and CsoR are both bacterial copper-responsive metalloregulators, yet they show distinct structural and mechanistic features revealed through ESR spectroscopy. These contrasting behaviors highlight the differences between transcriptional activators and repressors. In both *E. coli* and *P. aeruginosa*, CueR exists as a stable dimer in solution that does not rely on oligomeric transitions for function. Instead, the activation of transcription occurs through dynamical and conformational changes, particularly in the DNA-binding domain. CueR contains a canonical helix-turn-helix DNA-binding motif that enables interaction with DNA upon binding Cu(I), enabling RNA polymerase to bind and activate transcription. These changes occur without a change to oligomeric state, supporting an allosteric mode of activation without the need for major reassembly.

Although both CueR homologs share this dimeric structure, the responses to Cu(I) differ in *E. coli* and *P. aeruginosa* CueR. *E. coli* CueR shows an immediate increase in dynamics at the DNA-binding domain even at low Cu(I) concentrations, reflecting a rapid allosteric mechanism. This finding was supported by conformational changes observed through DEER spectroscopy, which revealed a squeezing motion of the DNA-binding domain upon Cu(I) coordination. In contrast, *P. aeruginosa* CueR exhibits gradual changes in dynamics that are tightly coupled to DNA binding rather than Cu(I) coordination. The difference can be explained by the presence of additional copper resistance mechanisms in *P. aeruginosa* not found in *E. coli*. With alternative pathways to mediate metal ion stress, *P. aeruginosa* CueR may require higher Cu(I) concentrations for activation, reflecting a more gradual response. These findings highlight the importance of studying protein dynamics to fully understand how homologous metalloregulators function in their specific cellular environments.

In contrast to the CueR activator mechanism, CsoR reveals a dynamic equilibrium between dimeric and tetrameric forms in solution. The relative populations of these states are sensitive to treatment with Triton X-100 and spin dilution, even in the absence of DNA or Cu(I), indicating that the oligomeric assembly itself is a critical feature of the regulatory mechanism of CsoR. As a transcriptional repressor, CsoR binds DNA in the metal-free state to inhibit transcription of Cu(I) regulatory genes. The results here indicate that DNA binding does not significantly shift the dimer-tetramer equilibrium, but it may alter the exchange between subunits in the oligomeric states. Small differences in subunit exchange in the presence of DNA might further fine-tune the protein-DNA interaction, possibly facilitating dissociation upon Cu(I) binding. This mechanism would support the model in which transcription repression by CsoR is connected to a higher-order oligomeric state.

More broadly, these differences in mechanisms between activators and repressors may indicate an evolved response based on the bacteria. Activators, like CueR, use conformational and dynamical flexibility to fine-tune the transcription of Cu(I) regulatory genes. On the other hand, repressors like CsoR rely more heavily on oligomerization, and the exchange of subunits in an oligomer may contribute to facilitating the transcription of DNA. Notably, CsoR proteins are the only known copper regulators in several bacterial genera and are widely distributed in organisms in which CueR is absent, suggesting that CsoR may have evolved as a solution for copper homeostasis in certain bacteria (114).

Finally, it is important to note that the experiments discussed here were performed *in vitro* using purified components, and the insights should be viewed in this context. The intracellular environment is more complex, with additional factors such as metal chaperones, reduction conditions, and macromolecular crowding likely influencing the protein. While the results discussed here provide a framework for understanding Cu(I) regulation in bacteria, in-cell ESR measurements, while technically difficult, will be valuable for refining these results under more native conditions. Overall, this work highlights how ESR spectroscopy provides insights into transcriptional regulation by resolving dynamical changes inaccessible by other biophysical techniques.

## Data and code availability

All data shall be made available if requested.

## Acknowledgments

S.S. and S.R. acknowledge the support from the 10.13039/100000001National Science Foundation-Binational Science Foundation (NSF-BSF, BSF no. 2018029, NSF no. MCB-2006154, BSF no. 2019723, and BSF no. 2022103).

## Declaration of interests

The authors report no competing interests.
